# Colour, Luminance and Crowding

**DOI:** 10.1068/ig1

**Published:** 2013-10-01

**Authors:** BJ Jennings, R Chakravarthi, J Martinovic

**Affiliations:** University of Aberdeen, UK; University of Aberdeen, UK; University of Aberdeen, UK

## Abstract

Three experiments were performed to assess the effect backgrounds have on object discrimination. Experiment 1 investigated the discrimination of foveally presented Gaborised objects and non-objects with and without a surrounding background. Thresholds were obtained by modulating the Gabor patches in 7 different directions, either isolating the L-M, S-(L+M) and L+M geniculate mechanisms, or stimulating these mechanisms in combination. The spacing between background Gabor elements and the object contour was chosen so as to not cause crowding, on the basis of previously published work with luminance stimuli. No differences were found between the Michelson contrasts required for threshold with or without a background, except when signals in the S-(L+M) and L+M were combined. The signals were combined at an elevation of 30° in DKL colour space, which resulted in a mixture with a proportionally strong chromatic signal. Experiment 2 investigated this finding further using three background conditions: no background, a sparse background and a densely populated background. Object vs. non-object discrimination thresholds were obtained for the L+M and S-(L+M) isolating directions, along with two conditions that combined them at DKL luminance elevations of 30° and 60°. In the 60° combination, the proportion of the chromatic signal was lower than in the 30° combination. Thresholds were found to be largely stable across chromatic and luminance conditions and background class, again with the exception of the combination at 30° elevation. The final experiment examined Gabor orientation discrimination over the same conditions as experiment 2 using a classical crowding paradigm, with a peripheral target and a set of three target-flanker separations. Crowding was most pronounced in the 30° combination. We conclude that when S-(L+M) signals above a certain level are combined with luminance signals, an increase in crowding results. This is likely to underlie the adverse effect of such signals on the discrimination of objects with backgrounds. [Research supported by BBSRC New Investigator grant to JM].

